# Effects of ghrelin supplementation on the acute phase of Chagas disease in rats

**DOI:** 10.1186/s13071-019-3787-y

**Published:** 2019-11-09

**Authors:** Ferdinando de Paula Silva, Cássia Mariana Bronzon da Costa, Luiz Miguel Pereira, Diego Fernando Silva Lessa, Dimitrius Leonardo Pitol, João Paulo Mardegan Issa, José Clóvis do Prado Júnior, Ana Amélia Carraro Abrahão

**Affiliations:** 1 Department of Clinical Analyses, Toxicology and Food Science, School of Pharmaceutical Sciences of Ribeirão Preto, University of São Paulo, Ribeirão Preto, 14040-903 Brazil; 2 Department of Morphology, Physiology and Basic Pathology, School of Dentistry of Ribeirão Preto USP, University of São Paulo, Ribeirão Preto, 14040-904 Brazil

**Keywords:** Chagas disease, Ghrelin, Immune response, Inflammation

## Abstract

**Background:**

*Trypanosoma cruzi* is the causative agent of Chagas disease, which is endemic to subtropical and tropical Americas. The disease treatment remains partially ineffective, involving therapies directed to the parasite as well as palliative strategies for the clinical manifestations. Therefore, novel candidates for disease control are necessary. Additionally, strategies based on parasite inhibition *via* specific targets and application of compounds which improve the immune response against the disease is welcomed. Ghrelin is a peptide hormone pointed as a substance with important cardioprotective, vasodilatory, anti-apoptotic, anti-oxidative and immune modulatory functions. The aims of this study were to evaluate the immunomodulatory effects of ghrelin in male Wistar rats infected with the Y strain of *T. cruzi*.

**Methods:**

In order to delineate an immune response against *T. cruzi* mediated by ghrelin, we evaluated the following parameters: quantification of blood and cardiac parasites; analysis of cell markers (CD3^+^, CD8^+^, NK, NKT, CD45RA^+^, macrophage and RT1B^+^); nitric oxide (NO) production; lymphoproliferation assays; splenocyte apoptosis; and INF-γ, IL-12 and IL-6 quantification in sera.

**Results:**

The animals infected with *T. cruzi* and supplemented with ghrelin demonstrated an upregulated pattern in macrophage and NO production, whereas an anti-inflammatory response was observed in T cells and cytokines. The low response against *T. cruzi* mediated by T cells probably contributed to a higher colonization of the cardiac tissue, when compared to infected groups. On the other side, the peptide decreased the inflammatory infiltration in cardiac tissue infected with *T. cruzi*.

**Conclusions:**

Ghrelin demonstrated a dual function in animals infected with *T. cruzi*. Further studies, especially related to the decrease of cardiac tissue inflammation, are needed in order to determine the advantages of ghrelin supplementation in Chagas disease, mostly for populations from endemic areas.

## Background

Chagas disease is a tropical neglected disease caused by *Trypanosoma cruzi*. The disease has an important socioeconomic impact in Latin America, afflicting about 6–8 million people whereas 65 million are under infection risk in endemic areas [1]. Due to the migration dynamics, the disease has been spread to non-endemic areas, such as North America, Europe, Japan and Australia [2]. In endemic areas, the disease is mostly transmitted by the contact with feces of infected triatomine insects after a bite or food contamination. Congenital transmission (including breastfeeding), blood transfusion organ transplantation and laboratory accidents are also important, particularly in non-endemic areas. Clinically, Chagas disease is divided into acute and chronic phases. The first is usually characterized by the absence or non-specific symptoms, evolving to an indeterminate form, which affects most of the infected patients. Some indeterminate patients develop the chronic phase, marked by cardiac (arrhythmia, heart-muscle disorder and heart failure) and/or digestive (enlargement of the oesophagus and the colon) disturbances [1].

Despite the efforts to develop new drugs and preventive approaches [3–6], there are only two available drugs (benznidazole and nifurtimox), which demonstrate side-effects and a limited anti-*T. cruzi* action [7]. Thus, novel forms to control the disease are welcomed. One of the control strategies is based on the administration of molecules related to immune modulation, which contributes to an effective response against the parasite or avoid the deleterious symptoms of the chronic phase. Ghrelin is a hormonal peptide produced in ghrelinergic cells in the gastrointestinal tract and performs an important role in the regulation of appetite and metabolism [8, 9]. The peptide stimulates the AMP-activated protein kinase (AMPK) in the hypothalamus, improving the glucose uptake, the fatty acid oxidation, glycolysis, whereas inhibits the fatty acid and glycogen synthesis and gluconeogenesis [10, 11].

Ghrelin also controls several immune functions, controlling inflammation and auto-immunity [12]. The mechanism of ghrelin immune regulation is based on the inhibition of the leptin and immune stimuli-induced pro-inflammatory cytokine production in T cells and monocytes, such as IL-1 beta, IL-6 and TNF-alpha [13]. Thus, the anti-inflammatory effects of ghrelin are observed in autoimmune encephalomyelitis [14], arthritis [15], sepsis [16], hepatic inflammation [17], colitis/inflammatory bowel disease [18], pancreatitis [19], gastritis [20], lung injury [21], myocardial infarction [22] and intestinal ischemia-reperfusion [23]. However, there are few studies for the use of ghrelin against infectious agents [20, 24]. Moreover, no studies have been performed in *T. cruzi* models, despite the potential of ghrelin for inflammation control. Therefore, there is a demand for the use of ghrelin in Chagas disease, which may contribute to alleviate the symptoms related to inflammation/auto-immunity, particularly cardiac lesion. Thus, our goal was to evaluate the immune effects of ghrelin administration during the acute phase of Chagas disease. The key points of the innate immune and adaptive responses, such as quantification of macrophages, NK/NKT cells, NO quantification, CD4^+^/CD8^+^ cells, T cell proliferation and apoptosis were evaluated. We also quantified the levels of cytokines (INF-γ, IL12 and IL-6) in serum, for a complete description of the anti-inflammatory response induced by ghrelin. Using these parameters, the positive and negative effects of ghrelin in animals infected with *T. cruzi* were determined and will base further strategies to describe the mechanisms related to Chagas disease pathogenesis/control.

The parameters from the acute phase are the first step to determine the potential of ghrelin as an immunomodulation agent for the control of Chagas disease symptoms. The anti-inflammatory pattern observed in the acute phase indicates the use of ghrelin for the chronic phase of Chagas disease, which probably contributes to reducing the intense cellular response and cardiac lesions [25]. Therefore, ghrelin has a potential to improve the immune response and alleviate the symptoms of Chagas disease, which will base a rational form to administrate this peptide to populations in endemic areas.

## Methods

### Animals, experimental infection and treatment

Male Wistar rats (90–100 g) were acquired from Facility House of the University Campus of Ribeirão Preto and housed two to a cage with water and feed *ad libitum*. Animals (6 per group) were randomized in the following groups: non-infected/non-treated control (C); non-infected/ghrelin treated control (CG); *T. cruzi* infected/non-treated group (I); and *T. cruzi* infected/ghrelin treated group (IG). Infected groups were intraperitoneally inoculated with 1 × 10^5^ blood trypomastigotes of the *T. cruzi* Y strain [26, 27], previously maintained by serial passage in Swiss mice. The ghrelin treatment was initiated 24 h after infection and performed for 14 days (single dose/day). We adopted a well-designed regimen of treatment, based on cardioprotective and anti-inflammatory studies performed in the rat model [28–33], which use ghrelin 100 µg/kg once or twice a day. Thus, ghrelin (R&D Systems, Tocris, USA) was diluted in saline and 100 µg/kg/day was administered *via* subcutaneous injection. On day 15 post-infection, the animals were anesthetized with isoflurane (2.5%) and euthanized by cervical dislocation. Spleens, peritoneal exudates and sera were collected for immune response evaluation and hearts were processed for histologic analysis.

### Parasitemia and animal weighing

Parasitemia was determined on day 8 post-infection using Brener’s method [34]. The animals were weighed on day 14 day post-infection using an electronic balance (Mettler-Toledo, Brazil).

### Peritoneal cells and oxide nitric

The peritoneal cells were collected and processed as described in [35]. Briefly, the cells were suspended in RPMI (Merck KGaA, Darmstadt, Germany) supplemented with fetal bovine serum (10%) and centrifuged at 1800×*g*, for 5 min at 4 °C. The pellets were suspended in ACK buffer (0.15 M NH_4_Cl, 1 mM KHCO_3_, 0.1 mM NaEDTA), incubated for 2 min at room temperature and centrifuged (1800×*g* for 5 min at 4 °C). The cells were suspended in RPMI and counted in a hemocytometer. The cell suspension was assessed for nitric oxide quantification and analysed by flow cytometry. For nitric oxide quantification [36], cells (100 µl, 2 × 10^5^ cells/well) were distributed in 96-well plates (Corning) and incubated with 1 µg/ml lipopolysaccharide (LPS, catalogue number: L2654. Sigma-Aldrich, Darmstadt, Germany) for 48 h, 37 °C, 5% CO_2_. After incubation, the cells were centrifuged at 1500×*g*, for 4 min, 20 °C and 100 µl of the supernatant transferred to a new 96-well plate. The samples were incubated with 100 µl Griess solution (1% sulfanilamide, 0.1% N-1-Naphthyl ethylenediamine in 5% phosphoric acid) for 15 min at room temperature. The plates were read at 540 nm in an ELISA reader (Synergy H1, Biotek, Winooski, VT, USA). The nitrite concentration was calculated by linear regression using a curve constituted by serial solutions of sodium nitrite (500 µM to 7.8 µM).

### Splenic cells

The splenic cell isolation was performed as described in [37]. Briefly, the cells from spleens were disaggregated mechanically using a 100 µm nylon cell strainer (Thermo Fisher Scientific, Pittsburgh, USA). The cells were washed with a hypotonic buffer (160 mM NH_4_Cl, 10 mM Tris-HCl, pH 7.4) and suspended in RPMI (2 × 10^7^ cells/ml). Cell viability was determined using a trypan blue assay (Sigma-Aldrich). The cells were assessed for phenotypic analysis, cell proliferation and apoptosis.

### Flow cytometry analysis

Splenic and peritoneal cells were processed similarly as described by [35]. The cell suspensions (100 µl/tube) were disposed in 12 × 75 mm round-bottomed polystyrene tubes (Falcon, USA) in staining buffer (BD-Pharmingen, San Diego, USA) and blocked using a Fc receptor blocking antibody (anti-CD32, BD-Pharmingen, San Diego, USA) for 20 min, at 4 °C in the dark. Peritoneal cells were labeled with anti-rat macrophage subset^+^/PE and/or anti-rat RT1B^+^/PerCP (catalogue numbers: 554901 and 557016, respectively. The splenic cells were incubated with the following monoclonal antibodies: anti-rat CD3^+^/APC (catalogue number: 557030), anti-rat CD4^+^/PECy-7 (catalogue number: 561578), anti-rat CD8^+^/PercP (catalogue number: 558824), anti-rat CD45RA^+^/PE (catalogue number: 551402), anti-rat CD161a^+^/FITC (catalogue number: 555008). All antibodies were purchased from BD Biosciences Pharmingen, CA, USA. The data acquisition was performed in a FACS Canto flow cytometer (BD Biosciences, California, USA) equipped with the FACSDiva software.

### *Trypanosoma cruzi* protein extract

For evaluation of the cell proliferation and apoptosis under determined stimulation, *T. cruzi* proteins extracts were obtained as described by Camargo et al. [38]. Trypomastigote forms (Y strain) were harvested from cells cultures (LLCMK2 lineage) and filtered with a 5 µm filter syringe. The parasites (1 × 10^8^/ml) were washed with phosphate-buffered saline (PBS) and suspended in RPMI. The suspensions were sonicated (two sonication cycles at 20 KHz, 30 watts in a QSonic sonicator, Cole Parmer, USA), centrifuged for 10 min, 10,000×*g*, 4 °C and the supernatant filtered with a 0.22 µm filter syringe. The protein concentration of the *T. cruzi* extract (supernatant) was determined using a Bradford protein assay (Pierce™ Coomassie/Bradford Protein Assay Kit, ThermoFisher Scientific, Waltham, USA). The concentration was then normalized to 3 mg/ml.

### T cell proliferation

The T cell proliferation was evaluated by a CFSE (carboxyfluorescein succinimidyl ester) assay, as described in [39]. The splenic cells (1 × 10^5^/tube) in RPMI were centrifuged (1500×*g*, 4 °C, 3 min) and suspended in 100 µl PBS. The cells were incubated with 5 µM carboxyfluorescein succinimidyl ester (CFSE, BD Biosciences Pharmingen, Franklin Lakes, USA) for 10 min, at 37 °C in the dark. The extracellular CFSE was blocked with RPMI supplemented with FBS (5%) and incubated for 5 min at room temperature. After incubation, the samples were centrifuged at 3500×*g* for 1 min at 4 °C and suspended in RPMI. The CFSE-labeled cells were divided into two groups. The first group (day 0) was incubated for 15 min, 37 °C, 5% CO_2_ and analyzed by flow cytometry. The second group was disturbed in 96-well plates and incubated for 4 days (day 4), at 37 °C and 5% CO_2_ with RPMI (control), 2.5 µg/ml Concanavalin A (ConA, Sigma-Aldrich) or 300 µg/ml *T. cruzi* protein extract. After incubation, the cells were transferred to 12 × 75 mm round-bottomed polystyrene tubes and washed three times with PBS. For tubes from days 0 or 4, the flow cytometry analysis was performed similarly. The samples were blocked with Fc receptor blocking (anti-CD32, BD-Pharmingen) for 20 min, at 4 °C in the dark. The cells were washed with PBS and labeled with anti-rat CD3^+^/APC (BD Biosciences Pharmingen, catalogue number: 557030). The decrease of CFSE intensity of the CD3^+^ population due to cell division was measured using CellQuest software (Becton Dickinson, San Jose, CA, USA).

### Apoptosis analysis (Annexin and PI)

The apoptosis evaluation was performed as described in [40]. The splenic cells (1 × 10^6^/ml) in 96-well plates (100 µl/well) were incubated for 24 h, 37 °C, 5% CO_2_ with *T. cruzi* extract (300 µg/ml) or RPMI (control). In parallel, the same approach was performed for 15 min. After incubation, the cells were transferred to 12 × 75 mm round-bottomed polystyrene tubes (100 µl/tube), washed twice with PBS and centrifuged for 3 min, 2500×*g*, 4 °C. The cells were suspended in Annexin-V FITC solution (1 µl Annexin-V FITC in 100 µl Annexin Binding Buffer, BD Biosciences Pharmingen) and incubated for 15 min, at room temperature, in the dark. Before the flow cytometry analysis, the cells received propidium iodide (PI, BD Biosciences Pharmingen). Early (Annexin^+^PI^−^) and late (Annexin^+^PI^+^) apoptosis were determined after analysis in a FACSCanto flow cytometer (BD Biosciences) equipped with the FACSDiva software.

### Cytokine quantification

The levels of IFN-γ, IL-12 and IL-6 were quantified using specific two-site enzyme-linked immunosorbent assay (ELISA) according to the manufacturer’s specifications and using reference standard curves. The samples (6 animals/group) were analyzed in duplicate and the plates read in an ELISA reader at 450 nm. All kits were purchased from RD Systems (DuoSet ELISA, Minneapolis, MN, USA).

### Cardiac parasite burden

The parasite burden in hearts was analyzed by counting after hematoxylin-eosin staining, as described in [41]. Briefly, hearts were immersed in 10% buffered formaldehyde and paraffin-embedded. The tissue was cut into 6 µm sections and stained with hematoxylin-eosin for evaluation of parasite burden and inflammation by optical microscopy. Parasite burden was estimated in sections separated at 70 µm intervals to avoid the recount of amastigote nests. The microscopic fields were analyzed at 400× magnification and all amastigote nests counted.

### Statistical analysis

Statistical analysis was performed using the program Graph Pad PRISM 5.0 (Graph Pad, USA). Data were analyzed using ANOVA followed by Tukeyʼs *post-hoc* test or unpaired Student’s t test (Figs. 9 and 10). All the results were expressed as mean with standard deviation. Differences were considered statistically significant at *P* < 0.05.

## Results

### Ghrelin decreased the weight of animals

**Fig. 1:**
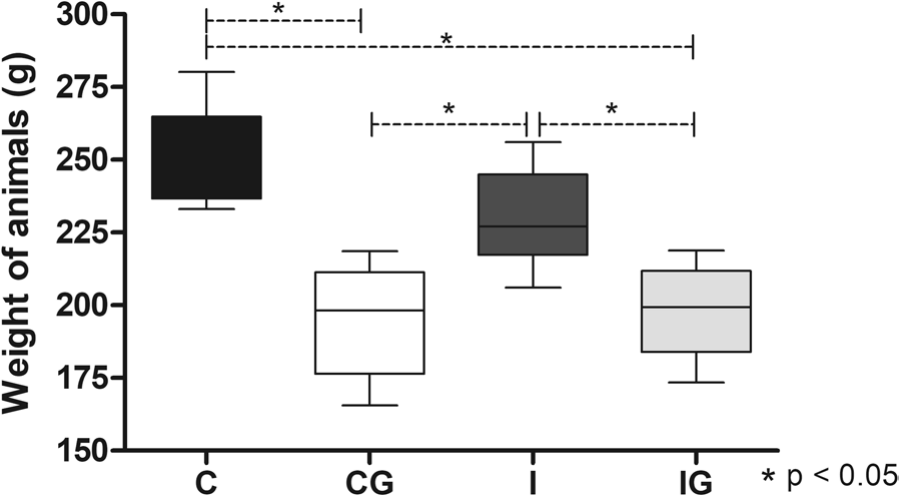
Weight of animals infected with *T. cruzi* and/or treated with ghrelin. Male Wistar rats were infected with *T. cruzi* and treated for 14 days with ghrelin. On day 15 post-treatment the animals were weighed in a balance and the results represented as grams (g). *Abbreviations*: C, non-infected and non-treated control; CG, non-infected and treated control; I, infected and non-treated animals; IG, infected and treated animals. *P < 0.05

The ghrelin supplementation significantly decreased (ANOVA: *F*_(3, 19)_ = 12.96, *P* < 0.0001) the weight of infected (IG) and non-infected (CG) animals, compared to the control (*post-hoc* tests: C *vs* CG, *P* = 0.0004; C *vs* IG, *P* = 0.0006) or the infected groups (*post-hoc* tests: I *vs* CG, *P* = 0.086; I *vs* IG, *P* = 0.0133) (Fig.1).

### Macrophage analysis and nitric oxide quantification

**Fig. 2:**
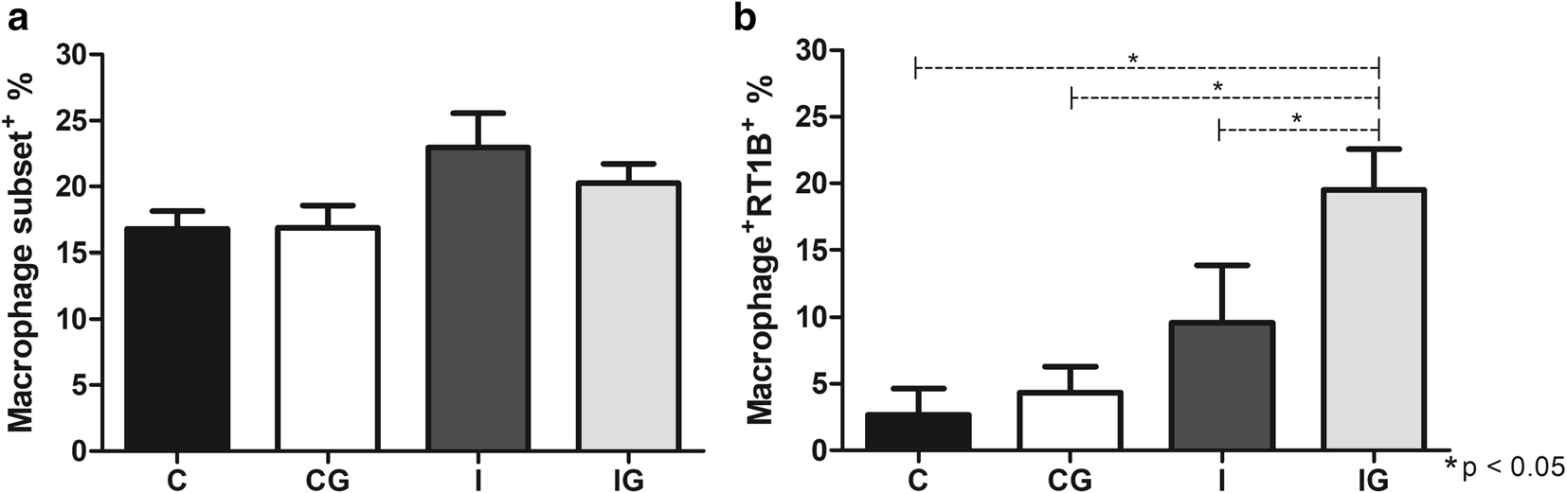
Analysis of peritoneal cells. Male Wistar rats were infected with *T. cruzi* and treated for 14 days with ghrelin. On day 15 post-infection, the animals were euthanized and the peritoneal cells collected. The cells were labelled with anti-rat macrophage subset^+^/PE and/or anti-rat RT1B^+^/PerCP antibodies and analyzed by flow cytometry. **a** Macrophage subset^+^ populations. **b** Macrophage^+^ RT1B^+^ populations. *Abbreviations*: C, non-infected and non-treated control; CG, non-infected and treated control; I, infected and non-treated animals; IG, infected and treated animals. *P < 0.05

**Fig. 3:**
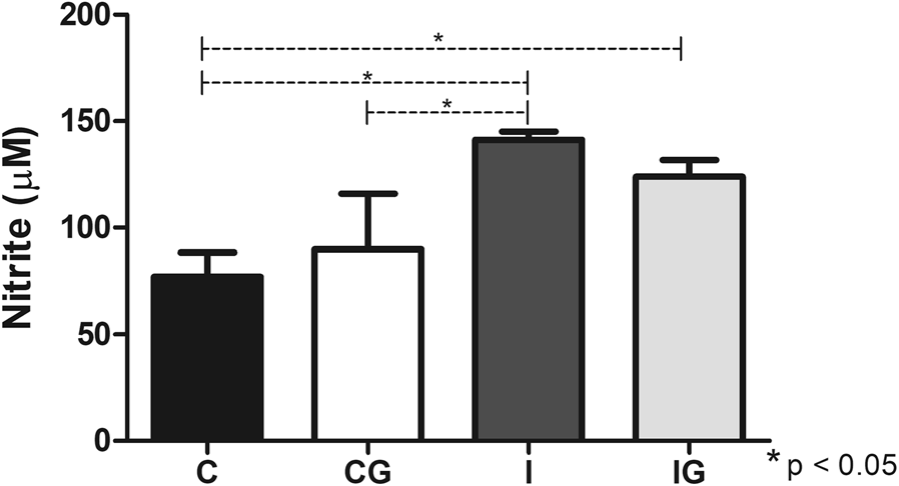
Nitric oxide quantification. Male Wistar rats were infected with *T. cruzi* and treated for 14 days with ghrelin. On day 15 post-infection, the animals were euthanized and the peritoneal exudate collected. Peritoneal cells (1 × 10^6^ cells/ml) were incubated with LPS for 48 h and the nitric oxide measured by Griess method, using serial dilutions of sodium nitrite as a reference. *Abbreviations*: C, non-infected and non-treated control; CG, non-infected and treated control; I, infected and non-treated animals; IG, infected and treated animals. *P < 0.05

The macrophage subset was not altered in animals infected with *T. cruzi* and/or supplemented with ghrelin (Fig. 2a). However, when macrophages that express MHC class II (RT1B^+^) were analyzed (ANOVA: *F*_(3, 15)_ = 27.56, *P* < 0.0001), the treatment with ghrelin (IG) significantly improved RT1B^+^ population in comparison to the infected group (*post-hoc* test: I *vs* IG, *P* = 0.0061), which was also elevated in relation to the control (*post-hoc* tests: C *vs* IG, *P* < 0.0001) and non-infected and treated animals (*post-hoc* test: CG *vs* IG, *P* < 0.0001) (Fig. 2b). The *T. cruzi-*infected animals (I) demonstrated an elevation (ANOVA: *F*_(3, 11)_ = 13.06, *P* = 0.0006) in nitric oxide production when compared to the control (*post-hoc* test: C *vs* I, *P* = 0.0003) and the non-infected and treated group (*post-hoc* test: CG *vs* I, *P* = 0.0214). The ghrelin supplementation in infected animals (IG) also improved the nitric oxide levels in relation to the control (*post-hoc* test: C *vs* IG, *P* = 0.0005), whereas non-significant alteration was observed compared to the infected animals (I) (Fig. 3).

### NK and NKT cells

**Fig. 4:**
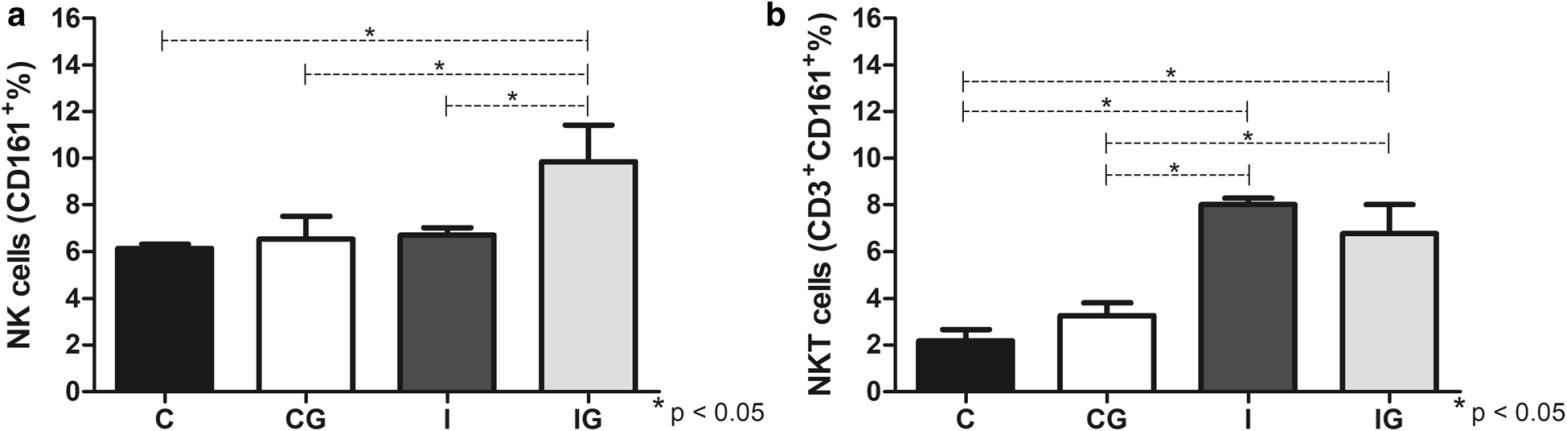
NK (CD161^+^) and NKT (CD3^+^CD161^+^) analysis. Male Wistar rats were infected with *T. cruzi* and treated for 14 days with ghrelin. On day 15 post-infection, the animals were euthanized and the spleens collected. The splenic cells were harvested and processed for NK (CD161^+^) and NKT (CD3^+^CD161^+^) detection. The cells were labelled with anti-rat CD161a^+^/FITC and/or anti-rat TCD3^+^/APC antibodies and analyzed by flow cytometry. **a** NK (CD161^+^) populations. **b** NKT (CD3^+^CD161^+^) populations. *Abbreviations*: C, non-infected and non-treated control; CG, non-infected and treated control; I, infected and non-treated animals; IG, infected and treated animals. *P < 0.05

The *T. cruzi* infection and ghrelin supplementation elevated the NK (ANOVA: *F*_(3, 16)_ = 16.77, *P* < 0.0001) and NKT (ANOVA: *F*_(3, 14)_ = 68.39, *P* < 0.0001) cells compared to the non-infected and non-treated (C) or the non-infected and treated (CG) counterparts (Fig. 4). The NK cells maintained a constant proportion among controls (C and CG) and the *T. cruzi* infected group (I), whereas the parameter significantly improved in the infected and treated animals (*post-hoc* tests: IG *vs* C, *P* = 0.0007; IG *vs* CG, *P* = 0.0038; IG *vs* I, *P* = 0.0023) (Fig. 4a). In contrast, the *T. cruzi* infection (I) elevated the proportion of NKT cells compared to the control groups (*post-hoc* tests: C *vs* I, *P* < 0.0001; CG *vs* I, *P* < 0.0001), which was maintained after ghrelin treatment (*post-hoc* tests: IG *vs* C, *P* < 0.0001; IG *vs* CG, *P* = 0.0002) (Fig. 4b).

### CD4^+^ and CD8^+^ analysis

**Fig. 5:**
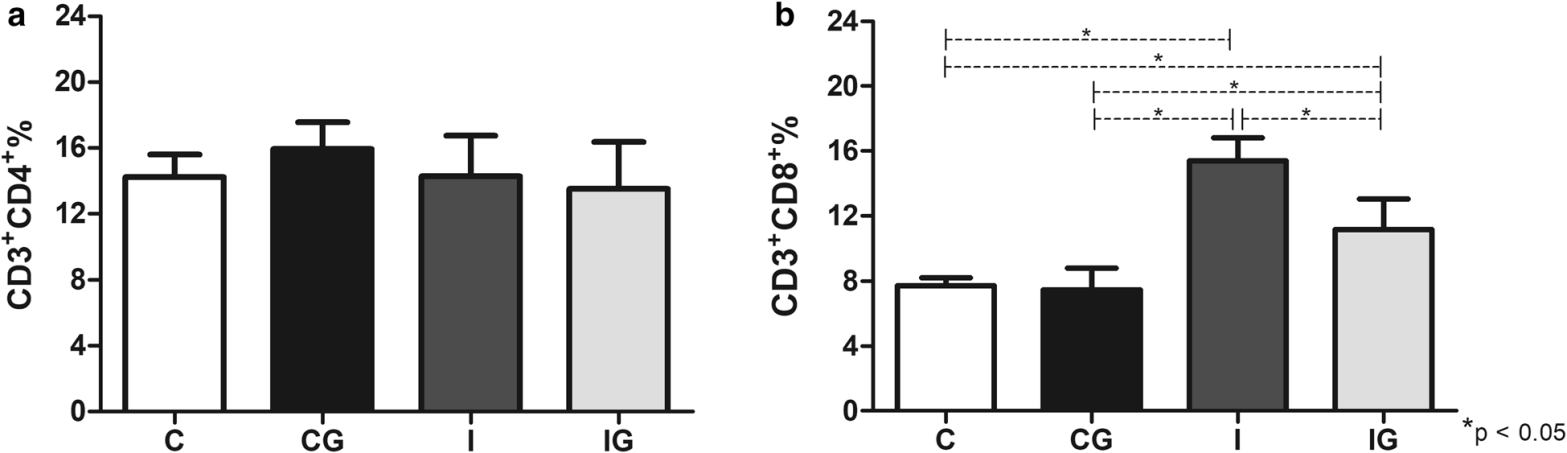
CD4^+^ and CD8^+^ populations. Male Wistar rats were infected with *T. cruzi* and treated for 14 days with ghrelin. On day 15 post-infection, the animals were euthanized and the spleens collected. The splenic cells were harvested and processed for CD4^+^ (CD3^+^CD4^+^) and CD8^+^ (CD3^+^CD8^+^) evaluation. For the T cell subset detection, the suspensions were labelled first with anti-rat CD3^+^/APC, followed by a specific separation using anti-rat CD4^+^/PECy-7 (TCD4^+^) or anti-rat CD8^+^/PercP (TCD8^+^). **a** CD4^+^ (CD3^+^CD4^+^) populations. **b** CD8^+^ (CD3^+^CD8^+^) populations. *Abbreviations*: C, non-infected and non-treated control; CG, non-infected and treated control; I, infected and non-treated animals; IG, Infected and treated animals. *P < 0.05

The CD8^+^ cells increased under *T. cruzi* infection (ANOVA: *F*_(3,15)_ = 38.69, *P* < 0.0001), whereas CD4^+^ cells were unresponsive, even after ghrelin treatment (Fig. 5). Treatment of infected animals with ghrelin significantly decreased the CD8^+^ cells (*post-hoc* test: I *vs* IG, *P* = 0.006); however, the population remained elevated when compared to the controls (*post-hoc* tests: IG *vs* C, *P* =0.0049; IG *vs* CG, *P* = 0.0105; I *vs* C, *P* < 0.0001; I *vs* CG, *P* < 0.0001) (Fig. 5b).

### T cell proliferation

**Fig. 6:**
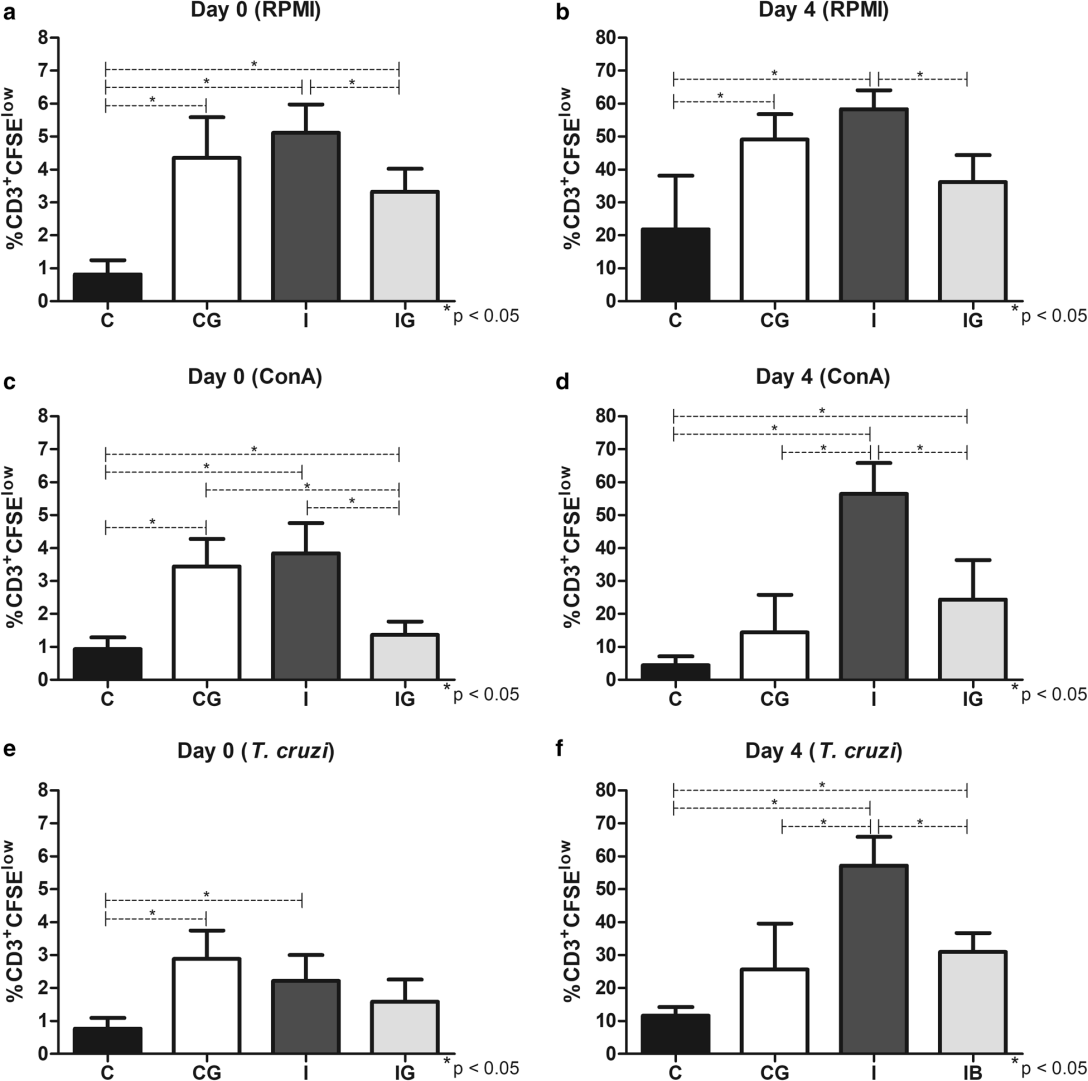
Evaluation of T cell proliferation by CFSE. Male Wistar rats were infected with *T. cruzi* and treated for 14 days with ghrelin. On day 15 post-infection, the animals were euthanized and the spleens collected. The splenic cells were processed, labelled with CFSE and incubated with ConA, *T. cruzi* extract or RPMI (control) for 4 days, 37 °C, 5% CO_2_. In parallel, the same approach was performed with incubation for 15 min (day 0). The T cell population was distinguished using anti-rat CD3^+^/APC antibody and the proliferation analyzed by flow cytometry. **a** Proliferation for 15 min (day 0) with RPMI. **b** Proliferation for 4 days (day 4) with RPMI. **c** Proliferation for 15 min (day 0) with ConA. **d** Proliferation for 4 days (day 4) with ConA. **e** Proliferation for 15 min (day 0) with *T. cruzi* extract. **f** Proliferation for 4 days (day 4) with *T. cruzi* extract. *Abbreviations*: C, non-infected and non-treated control; CG, non-infected and treated control; I, infected and non-treated animals; IG, infected and treated animals. *P < 0.05

In general, the T cells from infected animals proliferated under unspecific (ConA) and specific (*T. cruzi* extract) stimulation, mostly when incubated for 4 days (Fig. 6). The ghrelin treatment on the non-infected control (CG) indicated a pro-proliferative pattern compared to the non-supplemented counterpart (C). The cell proliferation of CG animals was elevated in cultures incubated for 15 min (ANOVA: *F*_(3, 18)_ = 26.66, *P* < 0.0001; *F*_(3, 16)_ = 4.59, *P* = 0.0168; and *F*_(3, 18)_ = 9.46, *P* = 0.0006 for Fig. 6a, c and e, respectively) in all conditions compared to the non-treated control (*post-hoc* tests: RPMI, *P* < 0.0001; ConA, *P* = 0.0002; and *T. cruzi* extract, *P* = 0.0004). When incubated for 4 days (ANOVA: *F*_(3, 16)_ = 11.94, *P* = 0.0002; *F*_(3, 15)_ = 20.95, *P* < 0.0001; and *F*_(3, 15)_ = 23.39, *P* < 0.0001 for Fig. 6b, d and f, respectively), the proliferation from CG groups was elevated only in the RPMI groups (*post-hoc* test: *P* = 0.0034), whereas in ConA and *T. cruzi* extract the proliferation was similar to the control (C) group (Fig. 6a, b, c and e). The infection with *T. cruzi* (I) improved the T cell proliferation in all conditions compared to the non-infected (C) control (*post-hoc* tests: the *P*-values for I *vs* C groups were *P* < 0.0001, *P* = 0.0002, *P* < 0.0001, *P* < 0.0001, *P* = 0.0092 and *P* < 0.0001 for a, b, c, d, e and f, respectively) (Fig. 6). The T cells from the infected group (I) were also responsive to specific (*T. cruzi* extract) and unspecific (ConA) stimuli (Fig. 6c–f). However, compared to the ghrelin-treated and non-infected groups (CG), the infection with *T. cruzi* (I) stimulated the T cell proliferation only the samples incubated with CoA (*post-hoc* test: *P* < 0.0001) and *T. cruzi* (*post-hoc* test: *P* = 0.0002) for 4 days (Fig. 6d and f). The treatment with ghrelin in *T. cruzi* infected animals (IG) indicated an antiproliferative pattern (*post-hoc* tests: the *P*-values for I *vs* IG groups were *P* = 0.0197, *P* = 0.0172, *P* = 0.0004, *P* = 0.0013, *P* = 0.0012 for Fig. 6a, b, c, d and f, respectively), except for the group incubated for 15 min under *T. cruzi* extract (Fig. 6e). However, the lower proliferation observed in IG animals in relation to infected ones (I) was superior when compared to the control (C) group (*post-hoc* tests: the *P*-values for IG *vs* C groups were *P* = 0.0008, *P* = 0.0425 and *P* = 0.0147 for Fig. 6a, d and f, respectively), except for cultures incubated with RPMI for 4 days and ConA and *T. cruzi* for 15 min (Fig. 6).

### Early and late apoptosis

**Fig. 7:**
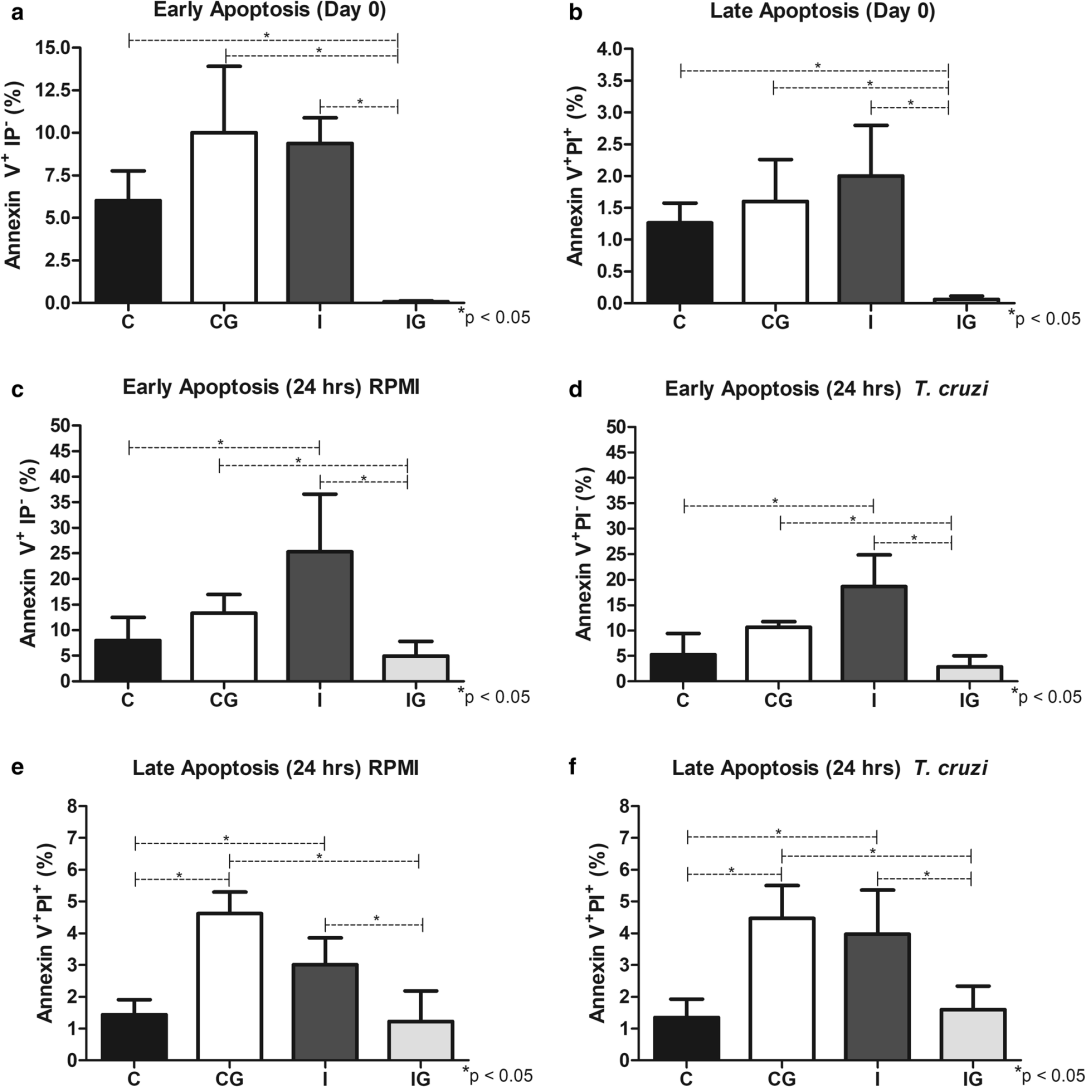
Early and late apoptosis of splenic cells. Male Wistar rats were infected with *T. cruzi* and treated for 14 days with ghrelin. On day 15 post-infection, the animals were euthanized and the spleens collected. The splenic cells were processed, labelled with CFSE and incubated with *T. cruzi* extract or RPMI (control) for 24 h, 37 °C, 5% CO_2_. In parallel, the same approach was performed with incubation for 15 min (day 0). The cells were incubated with Annexin-V FITC for 15 min, at room temperature in the dark. Before the analysis by flow cytometry, propidium iodide was added. **a** Early apoptosis (AnnexinV^+^PI^-^) of cells incubated for 15 min with RPMI. **b** Late apoptosis (AnnexinV^+^PI^+^) of cells incubated for 15 min with RPMI. **c** Early apoptosis (AnnexinV^+^PI^−^) of cells incubated for 24 h with RPMI. **d** Early apoptosis (AnnexinV^+^PI^−^) of cells incubated for 24 h with *T. cruzi* extract. **e** Late apoptosis (AnnexinV^+^PI^+^) of cells incubated for 24 h with RPMI. **f** Late apoptosis (AnnexinV^+^PI^+^) of cells incubated for 24 h with *T. cruzi* extract. *Abbreviations*: C, non-infected and non-treated control; CG, non-infected and treated control; I, infected and non-treated animals; IG, infected and treated animals. *P < 0.05

The effects of *T. cruzi* infection in the early and late apoptosis were observed only when cells were incubated for 24 h (ANOVA: *F*_(3, 14)_ = 9.07, *P* = 0.0014; *F*_(3, 12)_ = 12.01, *P* = 0.0006, *F*_(3, 15)_ = 18.92, *P* < 0.0001; and *F*_(3, 14)_ = 13.26, *P* = 0.0002 for Fig. 7c, d, e and f, respectively). In these groups, the infection improved both Annexin V^+^PI^-^ (early) and Annexin V^+^PI^+^ (late) populations compared to the non-infected/non-treated controls (*post-hoc* tests: the *P*-values for I *vs* C groups were *P* = 0.046, *P* = 0.0018, *P* = 0.0240 and *P* = 0.0033 for Fig. 7c–f). The high early and late apoptosis processes during *T. cruzi* infection were reverted after ghrelin supplementation in all conditions (*post-hoc* tests: the *P*-values for I *vs* IG groups were *P* < 0.0001, *P* < 0.0001, *P* = 0.0012, *P* = 0.0007, *P* = 0.0099 and *P* = 0.0139 for Fig. 7a–f, respectively). A similar pattern was observed when infected and treated animals were compared to the non-infected and non-treated counterparts (*post-hoc* tests: the *P*-values for IG *vs* CG groups were *P* < 0.0001, *P* = 0.0011, *P* = 0.042, *P* = 0.034, *P* < 0.0001 and *P* = 0.0033 for Fig. 7a–f, respectively). The apoptosis from animals treated with ghrelin (IG) was similar to the observed in the control (C). However, for cells incubated for 15 min (ANOVA: *F*_(3, 15)_ = 22.01, *P* < 0.0001; and *F*_(3, 19)_ = 12.06, *P* = 0.0001 for Fig. 7a and b, respectively), the early and late apoptosis from infected and treated groups (IG) was lower than the control (C) (*post-hoc* tests: the *P*-values of IG *vs* C groups were *P* = 0.0024 and *P* = 0.0097 for Fig. 7a and b, respectively). In non-infected animals, the ghrelin supplementation (CG) improved the late apoptosis in groups incubated for 24 h compared to the control (C) (*post-hoc* tests: the *P*-values of CG *vs* C groups were *P* < 0.0001 and *P* = 0.0007 for Fig. 7e and f, respectively).

### Cytokine quantification

**Fig. 8:**
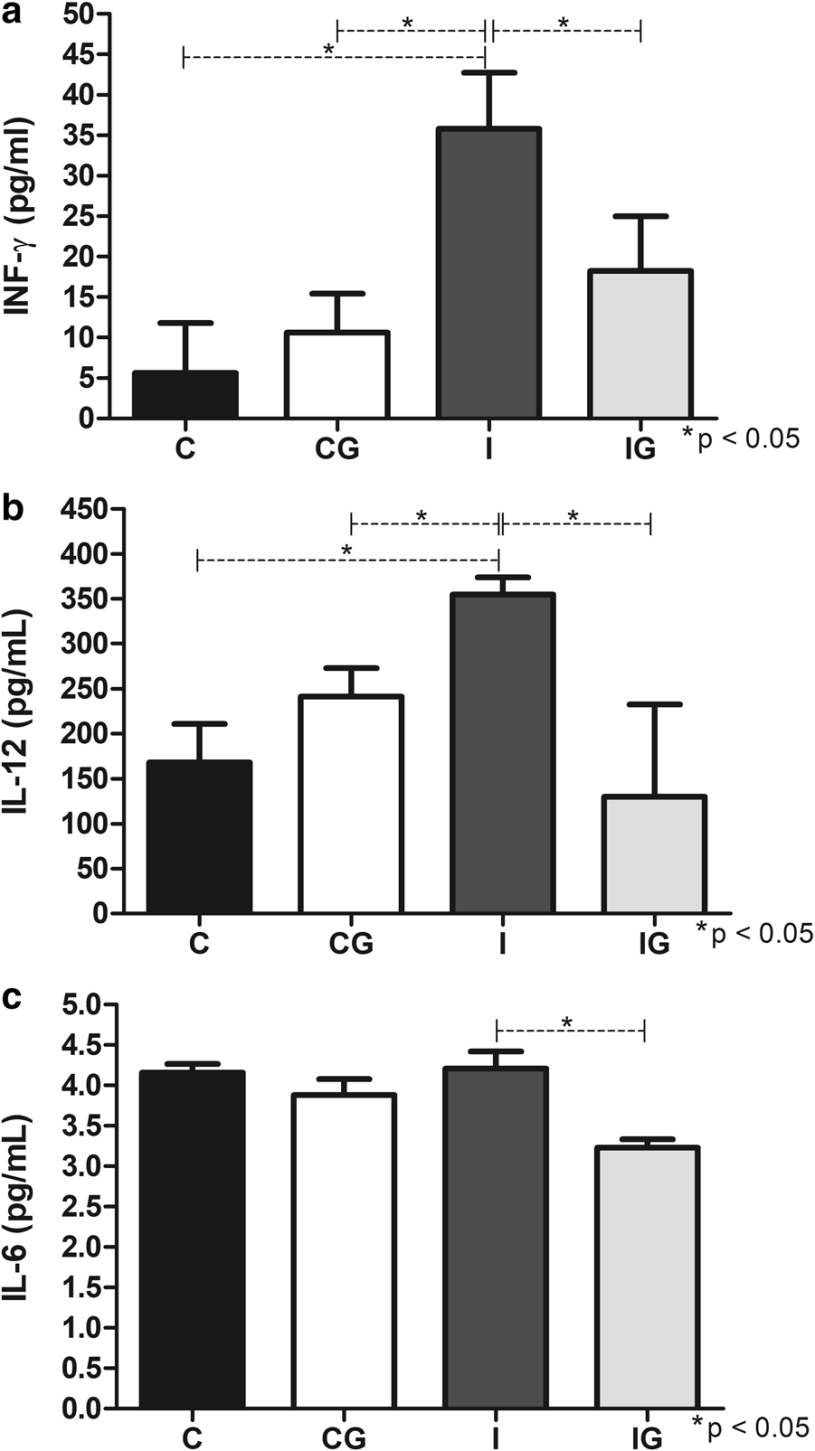
Cytokine quantification. Male Wistar rats were infected with *T. cruzi* and treated for 14 days with ghrelin. On day 15 post-infection, the animals were euthanized, and the sera collected. Cytokines INF-γ (**a**), IL-12 (**b**) and IL-6 (**c**) were quantified by ELISA. *Abbreviations*: C, non-infected and non-treated control; CG, non-infected and treated control; I, infected and non-treated animals; IG, infected and treated animals. *P < 0.05

The infection with *T. cruzi* elevated the levels of INF-γ (ANOVA: *F*_(3, 31)_ = 7.096, *P* = 0.0009) and IL-12 (ANOVA: *F*_(3, 16)_ = 10.20, *P* = 0.0005) compared to the non-infected and non-treated control (*post-hoc* tests: the *P*-values of I *vs* C groups were *P* = 0.0009 and *P* = 0.003 for Fig. 8a and b, respectively) or the non-infected and treated control (CG) (*post-hoc* test : the *P*-values of I *vs* CG groups were *P* = 0.0059 and *P* = 0.0414 for Fig. 8a and b, respectively). However, the *T. cruzi* infection did not alter the IL-6 levels (ANOVA: *F*_(3, 16)_ = 10.61, *P* = 0.0004) compared to the non-treated controls (Fig. 8c). The treatment with ghrelin in infected animals decreased all cytokine levels (*post-hoc* tests: the *P*-values of IG *vs* I groups were *P* = 0.032, *P* = 0.0005 and *P* = 0.0007 for Fig. 8a, b and c, respectively) and no alteration was observed when non-infected controls (CG) were treated (Fig. 8).

### Parasitemia and cardiac parasite burden

**Fig. 9:**
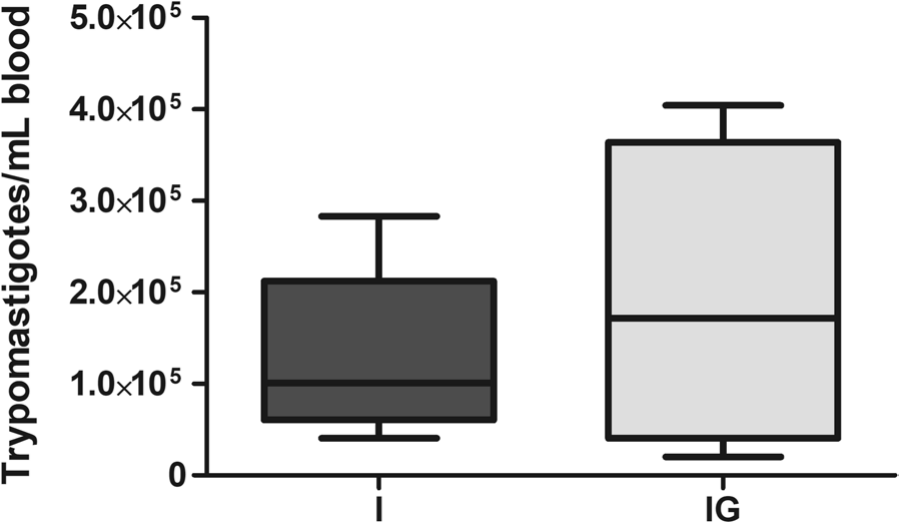
*T. cruzi* blood parasitemia. Male Wistar rats were infected with *T. cruzi* and treated for 14 days with ghrelin. On day 8 post-infection, blood was collected and the trypomastigotes forms counted according to Brener’s method

**Fig. 10:**
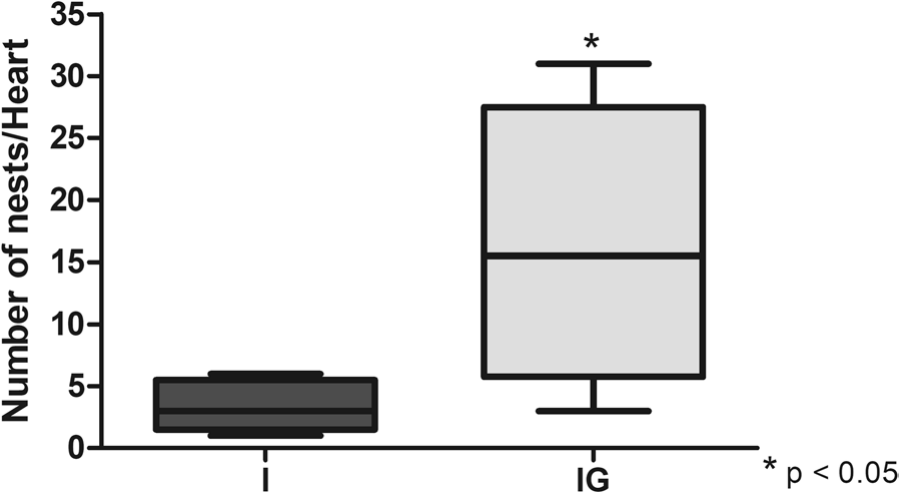
Cardiac parasite burden. Male Wistar rats were infected with *T. cruzi* and treated for 14 days with ghrelin. On day 15 post-infection, the animals were euthanized, and the hearts collected. The cardiac tissue was prepared and hematoxylin-eosin stained for detection and counting of amastigotes nests. *P < 0.05

**Fig. 11:**
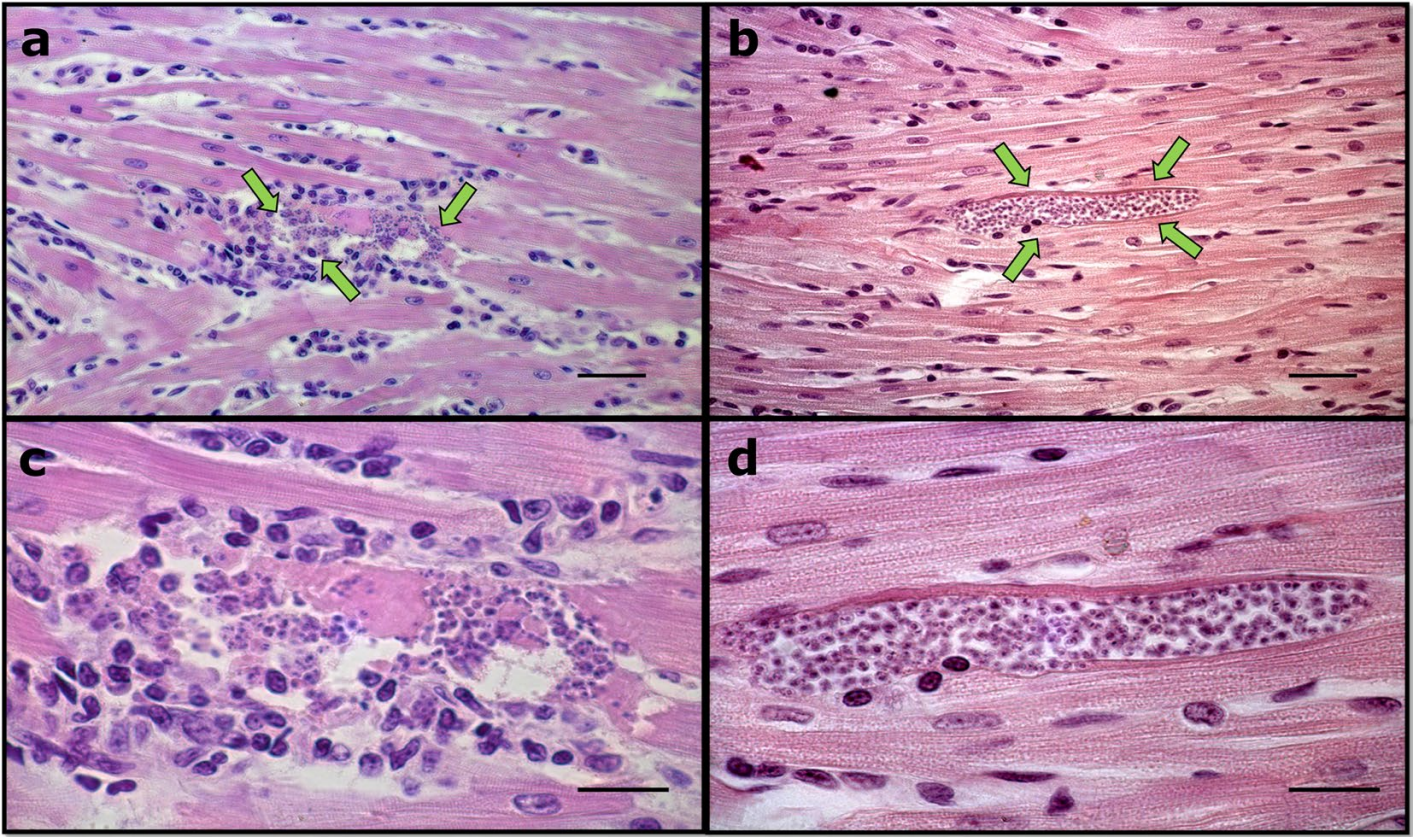
Colonization of cardiac tissue with *T. cruzi* amastigotes. Male Wistar rats were infected with *T. cruzi* and treated for 14 days with ghrelin. On day 15 post-infection, the animals were euthanized, and the hearts collected. The cardiac tissue was fixed with 10% buffered formaldehyde, paraffin-embedded and prepared for hematoxylin-eosin staining. Arrows indicate the amastigote nests. **a** Cardiac tissue from infected and non-treated animals (400×). **b** Cardiac tissue from infected and ghrelin-treated animals (400×). **c** Cardiac tissue from infected and non-treated animals (1000×). **d** Cardiac tissue from infected and ghrelin-treated animals (1000×). *Scale-bars*: **a**, **b** 100 µm; **c**, **d**, 50 µm

The treatment with ghrelin did not alter the blood parasitemia on day 8 post-infection (Fig. 9). In hearts (t-test: *t*_(7)_ = 2.458, *P* = 0.0063) the treatment with ghrelin elevated the number of amastigotes nests compared to the infected and non-treated group (I *vs* IG, *P* = 0.0419) (Fig. 10). However, when amastigotes nests were analyzed by microscopy, the treatment with ghrelin (IG) demonstrated a lower presence of inflammatory infiltration (Fig. 11b, d) in relation to the *T. cruzi-*infected group (I) (Fig. 11a, c).

## Discussion

A complex cellular and humoral immune response is mobilized against *T. cruzi* infection, performing an import role in the parasite-host relationship [42, 43]. During the acute phase of Chagas disease, the trypomastigotes forms of *T. cruzi* are abundant in peripheral blood [44] and the transition to the chronic stage is characterized by parasite migration to tissues (e.g. heart, colon and oesophagus) concomitantly with a specific immune response. The deleterious symptoms of the chronic phase are the result of a discomposed immune response against the intracellular amastigotes, which usually result in inflammation and tissue lesions. Once ghrelin has an anti-inflammatory pattern in diverse models [45], a set of assays are necessary to determinate the ghrelin effects during Chagas disease.

The pro-metabolic effect of ghrelin is observed in the rat model. The peptide administration decreased the animal weight, a consequence of the use of ghrelin in the control of obesity and metabolic-related disorders [46]. The weight loss verified in ghrelin-treated animals was not influenced by *T. cruzi* infection, which indicates the use of ghrelin as a metabolic regulator in endemic areas [47]. However, restrictions should be considered for the correct use of ghrelin in models also affected by malnutrition, which may lead to aggravation of Chagas disease symptoms. Indeed, authors have correlated the aggravation of *T. cruzi* infection with deprivation of nutrients [48]. For obese models infected with *T. cruzi*, ghrelin probably has a dual effect. Besides the weight loss, ghrelin decreases the TNF, IL-17 and IL-12p40 mRNA expression in the hearts of obese rats [10].

The initial phase of *T. cruzi* infection triggers several mechanisms of the innate response, such as nitric oxide production and activation of antigen-presentation (APCs) and NK cells [49]. The nitric oxide (NO) produced by macrophages has a pivotal anti-*T. cruzi* effect also observed in several infections caused by viruses, bacteria, fungi and protozoans [50]. The *T. cruzi* infection elevated the NO production, which is maintained after ghrelin administration. A constant and regulated NO production is essential to control the infection as well as avoiding tissue damage due to NO overproduction [51]. The macrophage is closely related to NO production and antigen presentation during infections [52]. Therefore, a specific antigen presentation is fundamental for an effective parasite control. Ghrelin promoted the expansion of MHC II presenting macrophages (RT1B^+^), which are important for an adequate adaptive response against *T. cruzi* [53]. The host resistance against the acute phase of Chagas disease is also dependent on NK and NKT cells, which produce pro-inflammatory cytokines and stimulates the phagocytic activity [54, 55]. Ghrelin acts by improving the NK and NKT cells in infected animals, maintaining an anti-*T. cruzi* pattern. Indeed, *in vitro* incubation of peripheral blood from pregnant women with ghrelin elevates the NK cells proportion [56]. However, more studies are required for the complete elucidation of the ghrelin mechanisms on NK/NKT cells during Chagas disease.

Despite the positive effects of ghrelin supplementation in innate response, the administration of the peptide decreased the T cell populations, mostly CD8^+^. The CD8^+^ mediated response controls infections caused intracellular protozoans such as *Plasmodium*, *Toxoplasma*, *Leishmania* and *Trypanosoma* [57]. Thus, this cell population has an important role in amastigote elimination in infected tissues. However, the typical cardiac lesions observed in the chronic phase of Chagas disease have been related to inflammatory infiltrates, which are mainly composed by CD8^+^ cells [58]. During the early stages of *T. cruzi* infection, parasite antigens are presented by antigen-presenting cells (APCs) using the major histocompatibility complex (MHC I or MHC II). In lymphoid organs, MHCs interact with the T cell receptor (TCR) and costimulatory molecules (CD80, CD86, CD40 and CD83), resulting in activation and proliferation of naïve T cells [59]. T cells (CD4^+^ and CD8^+^) perform an important role in the parasite control, producing cytokines (e.g. IFN-γ, IL-6 and IL-12) and factors for an effective cellular and humoral response [25]. Indeed, the levels of IFN-γ, IL-6 and IL-12 were lower after the treatment of infected animals with ghrelin. The *T. cruzi* infection stimulated the proliferation of CD3^+^ cells, which was reverted after ghrelin treatment. Moreover, the low proliferation of T cells was followed by the elevation of parasite colonization in the heart. However, ghrelin improved the cell proliferation in non-infected cells, indicating different mechanisms in infected and healthy models. To elucidate the ghrelin mechanisms in T cell activation, more assays related to Th1 and Th2 response and regulation are necessary, once some of the deleterious effects of the disease are related to an exacerbated CD8^+^ mediated response [60]. The T cell activation and expansion during Chagas disease is also followed by the improvement of apoptosis [61]. As a survival mechanism, *T. cruzi* produces several factors which induce the cell apoptosis and, consequently, parasite propagation. For example, the trans-sialidase enzymes, which are related to the caption of exogenous sialic acid residues from the host to the *T. cruzi* surface, induce an intense process of apoptosis [62, 63]. Ghrelin acts as a potent anti-apoptotic molecule during the acute phase of Chagas disease, decreasing the Annexin^+^ and/or PI^+^ cells in all tested conditions. Indeed, ghrelin inhibits apoptosis processes in diverse cellular models [64–66], indicating its cell protective pattern. Therefore, there is a potential for the ghrelin use during the chronic phase of Chagas disease, which may decrease the damage of infected organs induced by apoptosis [67, 68].

Despite the pivotal role of NK cells and macrophages in the secretion of INF-γ and IL-12 [69], our results indicate a reduction of these cytokines in animals treated with ghrelin. NK/NKT cells usually produce INF-γ in the early stages of infections, before the establishment of an adaptive response [70]. IL-12 is produced by antigen-presenting cells (APCs), including macrophages [71]. This cytokine stimulates T cell differentiation and proliferation, which also secretes INF-γ [72, 73]. Additionally, the pro-inflammatory IL-6 cytokine was also decreased in infected and ghrelin treated group. IL-6 stimulates specific differentiation of naïve T CD4 cells, a crucial step for the development of an acquired immune response [74]. The cytokine also stimulates the T cell proliferation under inflammatory conditions [75], leading to spontaneous rheumatoid arthritis (RA)-like disease in mice [76].

Once ghrelin decreased CD8^+^ populations and the CD3^+^ cell proliferation in infected animals, the low levels of INF-γ, IL-12 and IL-6 are probably related to the T cell suppression. Thus, Ghrelin promoted the innate response against *T. cruzi* and failed at promoting some points of T cell response (low CD8^+^ and T cell proliferation), which probably are related to the improvement of cardiac parasite burden. However, the anti-inflammatory pattern (INF-γ, IL-12 and IL-6) observed after ghrelin treatment is related to the lower presence of inflammatory infiltrates compared to the infected control. Indeed, in the chronic phase of Chagas disease, when parasite burden is usually controlled by the immune system [77], some patients demonstrate an intense inflammatory response in the cardiac tissue, mainly caused by CD8^+^ cells [78]. Moreover, INF-γ and IL-6 are overproduced in lymphocytes of patients with chronic chagasic cardiopathy (CCC) compared to asymptomatic individuals [79, 80], thus reinforcing the anti-inflammatory propriety of ghrelin.

## Conclusions

The use of ghrelin during the acute phase of Chagas disease demonstrated a dual pattern. The peptide promoted an effective innate response; however, several aspects of cell response are downregulated. Once ghrelin decreases the pro-inflammatory cytokines in serum and cell infiltrates in infected hearts, the peptide has the potential to control the main injuries caused by Chagas disease. A positive effect of ghrelin as cardio-protector, as observed in other models [30, 81, 82], will be important for therapies directed toward the alleviation of Chagas disease symptoms, improving the life quality of patients.

## Data Availability

All data generated or analyzed during this study are included in this published article.

## Abbreviations

CD: cluster of differentiation
NK: natural killer cell
NKT: natural killer T cell
NO: nitric oxide
IFN-γ: interferon gamma
IL: interleukin
RPMI: Roswell Park Memorial Institute media
FBS: fetal bovine serum
ACK: ammonium-chloride-potassium
LPS: lipopolysaccharides
APC: antigen-presenting cell
CFSE: carboxyfluorescein succinimidyl ester
ConA: concanavalin A
